# A Case of Transient Global Amnesia: A Rare Diagnosis

**DOI:** 10.7759/cureus.21637

**Published:** 2022-01-26

**Authors:** Naresa S Ramjohn, Abubaker Kallan, Mohammed A Qureshi

**Affiliations:** 1 Internal Medicine, Saint James School of Medicine, Chicago, USA; 2 Internal Medicine, Avalon University School of Medicine, Willemstad, CUW; 3 Internal Medicine, MacNeal Hospital, Berwyn, USA

**Keywords:** neocortex, hippocampal cortex, memory, covid-19, anterograde amnesia, retrograde amnesia, amnesia, memory loss, transient ischemic attack, transient global amnesia

## Abstract

Transient global amnesia (TGA) is a syndrome characterized by a loss of anterograde memory with a less prominent loss in retrograde episodic memory that resolves within 24 hours or less. In this report, we present a rare case of a 62-year-old male who presented to the emergency department with sudden onset confusion and memory loss. Prior to this, the patient had no significant medical or psychiatric history. Magnetic resonance imaging (MRI) and computerized tomography (CT) showed a normal presentation, and a neurology consultation ruled out any organic brain abnormalities. After ruling out all other potential causes, diagnosis of transient global amnesia was made. We present this case highlighting the importance of ruling out other acutely morbid conditions when addressing TGA, guidance on timing of imaging, as well as offering insight on other etiologies of this condition.

## Introduction

Transient global amnesia (TGA) is a rare clinical syndrome with an incidence of 5.2-10/100,000 per year in the general population and 23.5-32/100,000 in individuals greater than 50 years old [1]. TGA commonly presents in the seventh decade of life with the mean age range from 61 to 67.3 years, according to Spiegel et al. [2]. This syndrome is characterized by a loss of anterograde memory, that is, the inability to form new memories, with a less prominent loss in retrograde episodic memory which includes recognition and executive function [2]. This syndrome is demonstrated by a sudden disturbance in memory [3] which resolves in 24 hours or less [4]. During an attack, patients are unable to encode new information and transform it into long-term memory, therefore, they repeatedly ask questions about their surroundings [3]. Less prominently, but still present, patients display retrograde amnesia and are therefore also unable to recall information that occurred hours, days, months, or even years before the attack [3]. Here, we present a rare case of TGA in this study.

## Case presentation

A 62-year-old male presented with the chief complaint of confusion and memory loss that occurred at 9:50 AM, while at work. At the time of the episode, the patient was working his regular job at a fiberglass factory with his son and cousin. While on his break, he suddenly became confused and began repetitively asking, “where are we?” and “what happened?” The patient had forgotten where he lived and who he was working with. He was unable to identify his cousin and confused him for his son. Prior to this episode, his behavior was noted to be normal. At 12:04 PM, he presented to the emergency department (ED) with his son, who was the primary historian and translator for the patient. Upon being questioned, he was unable to report the last thing he could remember. There was no head injury, limb weakness, paralysis, changes in vision, headache, chest pain, shortness of breath, nausea, vomiting, or any other physical complaints at the time, but it was noted to be a hot day. The rest of the review of systems was insignificant. There was also no history of traumatic brain injury, cerebrovascular accidents, dementia, seizures, psychiatric illnesses, or previous similar episodes. Upon initial evaluation in the ED, there was no obvious facial asymmetry or unilateral weakness, and the blood glucose level was 100 mg/dL. On a mini-mental status examination (MMSE), the patient was unable to answer the current year, president, or home address. The patient did not have any past medical history, similar episodes of memory loss, and currently, he was not on any medications. Surgical history was significant for unspecified foot surgery in the remote past. The patient denied any tobacco, alcohol, or recreational drug use. 

On the physical examination in the ED, the patient’s blood pressure was 162/81 mmHg, pulse was 71 beats per minute, temperature was 98.4°F, respiration was 18 breaths per minute, and oxygen saturation was 96% on room air. Head, eyes, ears, nose, and throat (HEENT), cranial nerve examination, and sensation and strength in all extremities were all within normal limits, in addition to the neurological examination which demonstrated a conscious, coherent, and alert patient with no focal deficits and a normal gait. The rest of the physical examination was also insignificant. Glasgow Coma Scale eye subscore was 4, verbal subscore was 5, and motor subscore was 6. The patient was negative on the Cincinnati Prehospital Stroke Scale and was alert and oriented to person but not to place or time. 

On admission, laboratory studies including complete blood count (CBC), complete metabolic panel (CMP), prothrombin (PT), activated prothrombin time (aPTT), lipids, thyroid-stimulating hormone (TSH), coronavirus disease 2019 (COVID-19) polymerase chain reaction (PCR) screen and toxicology screen were unremarkable. During his hospital stay, the only medication that he received was enoxaparin 40 mg subcutaneous injection daily for deep vein thrombosis prophylaxis. A non-contrast computed tomography (CT) scan was also done approximately four hours after the episode, revealing no evidence of any intracranial abnormalities. Following this, a CT angiogram was done at the five-hour mark after the inciting event, which demonstrated no evidence of any vessel occlusion, stenosis, or aneurysm. In addition, T1-weighted, T2-weighted, diffusion-weighted, and fluid-attenuated inversion recovery (FLAIR) magnetic resonance imaging (MRI) was obtained eleven hours after the event. All were insignificant except for the T2-weighted and FLAIR images which demonstrated non-specific white matter changes and an incidental finding of an arachnoid cyst in the posterior fossa compared to the non-contrast CT. 

On evaluation 24 hours since the incident, the patient was oriented to person and place but not time. By this point, the patient had improved with minimal to no interventions. Electroencephalography (EEG) conducted 56 hours after the incident, demonstrated a background rhythm of 8-10Hz, no focal slowing, and no seizure activity. Upon discharge, 58 hours after the event, the patient was alert and oriented to person, place, and time and had returned to baseline. A follow-up visit was planned for one week later.

## Discussion

In 1990, Hodges and Warlow established the clinical criteria for TGA and this is still used today for clinical diagnosis (Table 1) [2,5]. We present a case that fits the established criteria. Our patient fell within the general age demographic for most reported cases of transient global amnesia which is between 61 and 67.3 years but had no other definite characteristics or risk factors [1]. When this episode occurred, he was not in his home, but at his place of work where stress levels are usually higher. His son had also reported that the patient had a rigorous schedule working two jobs, one after the other every day leading us to believe stress may have played a role. Further supporting this theory, a report from a neurological emergency department in a German academic hospital described an upsurge in patients with TGA thought to be due to increased stress levels from the severe acute respiratory syndrome coronavirus 2 (SARS-CoV-2) pandemic. An intake of 16 new patients with TGA was reported from February 1, 2020, to May 15, 2020, compared to an average number of just 9.7 that they received over the previous 10 years in total implying that social distancing, uncertainty of the future, and generalized fear may be contributing to increased stress levels leading to a greater incidence in TGA [6]. We can only speculate that stress levels may have played a role in the event that led to his confusion.

**Table 1:** Hodges and Warlow criteria for TGA TGA: transient global amnesia

Hodges and Warlow Criteria for TGA
Attacks must be witnessed from a capable observer
There must be anterograde amnesia during the attack
There must be no clouding of consciousness or loss of identity
Cognitive impairment must be limited to amnesia (no apraxia or aphasia, etc.)
There must be no focal neurological signs and symptoms before or after the attack
There must be no epileptic features
Attacks must be resolved within 24 hours
There must be no recent head injury or active epilepsy

To aid in the diagnosis, historically, magnetic resonance diffusion-weighted imaging (DWI) has been used to detect lesions associated with TGA [2]. Although this technique did not reveal anything significant in our patient, there has been reported evidence that shows activity in the hippocampal/entorhinal cortex network which involves the formation of early memory and its transference to remote memories stored in the neocortex [2]. The cornu ammonis (CA1) neurons is an area of focus because they are particularly vulnerable to stress [2]. Typically, unilateral lesions are found, and tend to be small (1-3mm) with high signal foci in the CA1 field of the hippocampus [2]. These lesions are prominently found 24-48 hours after the TGA attack [2]. Our patient had his MRI done 11 hours after the episode which did not demonstrate any significant findings. Although this is a diagnosis of exclusion, the MRI should be repeated at the minimum of 20 hours after onset as evidenced by Szabo et al. to aid in the diagnosis, especially if there is diagnostic uncertainty [7]. Including this in the diagnostic criteria would provide an additional step in diagnosing and understanding the pathophysiology of TGA. In doing so, clinicians can work towards the management of TGA and prevent episodes from happening in the first place. In a recent retrospective observational study done by Szabo et al., DWI was performed on 390 patients within one to three days of presentation to the hospital; 70.6% of patients with TGA had hippocampal DWI lesions [7]. Their cohort was missing relevant clinical information, however, having done an MRI helped to reinforce their clinical certainty. Therefore, in these situations, this diagnostic criterion becomes useful and adds to the body of knowledge of this disease. Their research has demonstrated the utility of these lesions in diagnosing TGA and imaging, which may indefinitely, play a huge role in the diagnostic criteria [7].

Ever since the recognition of this pathology by Morris Bender in 1956 and the conception of the term by Fisher and Adams two years later, the etiology of transient global amnesia has been widely hypothesized with no general consensus to date [8]. There have been many theories that have been up for debate as to why TGA occurs, but there has been no clear evidence to support one definitive cause [3]. Such theories include the disturbance of venous hemodynamics, epilepsy, migraines, and stress [3,2]. According to a study done by Szabo et al. in 2020, a potential precipitating event was evidenced in 61.1% of their cohort and 48.5% was emotional or physical stress that occurred immediately before the episode [7]. Another report observed that 90% of cases presented with a preceding event that was described as physical, emotional, or behavioral stress which implies that stress could play a main role in TGA [9]. A different study was done that demonstrated a prevalence of 80% of internal jugular venous valve insufficiency in 142 TGA patients compared to a prevalence of 25% in controls supporting the theory of Valsalva and venous hemodynamics [1]. Supporting the psychiatric theory, a study compared 51 subjects with psychiatric conditions (defined as depression or anxiety disorder) with TGA to 51 patients with TGA only with results showing an odds ratio of 2.86 in the patients with psychiatric disease [1]. In addition, a retrospective case-control study done by Jang et al. found that there was a higher incidence of hyperlipidemia among TGA patients compared to the matched controls [10]. Our patient did not display any of these co-incident health conditions but did experience this event during a stressful situation, in his work environment. Therefore, this emphasizes the awareness that needs to be made since TGA can present without any physical abnormalities or concomitant pathologies. 

Given that there is no universally accepted pathophysiology of TGA, there is also limited acceptance of a common treatment [2]. TGA’s short duration may also contribute to that idea. Treatment is mainly supportive with consideration of IV thiamine as a recommendation [1]. The patient is usually observed in the hospital until memory impairment resolves [1]. Besides a low dose of enoxaparin, our patient received little to no treatment during his hospital stay.

TGA is generally considered to be a rare, benign condition with a promising prognosis. A population-based, matched cohort study of TGA long-term risks among residents of Olmsted County, MN, from January 1, 1985, to December 31, 2010, found that the recurrence rate of TGA is estimated to be 2.9-23.8% [11]. In another 12-year follow-up study, TGA patients were compared to controls with endpoints of transient ischemic attack (TIA), CVA, seizures, and cognitive deficits with no statistically significant differences between the groups [2]. Although TGA is a pathology with a diagnostic criterion, it is also still regarded as a diagnosis of exclusion. We present a case with no definitive precipitating events, risk factors, comorbidities, and a normal clinical evaluation in all aspects. This report is an additional entry to the body of literature in this widely speculated pathology, to present an alternative perspective on approaching TGA cases. Highlighting the fact that despite the strong evidence supporting a multitude of etiologies, TGA may present in the generally healthy with few or no comorbidities, risk factors, or precipitating events. Clinicians should continue to place an emphasis on ruling out other acutely morbid conditions such as TIA, trauma, malignancy, and epilepsy, rather than seeking TGA, a generally benign condition. More research needs to be done investigating the multiple proposed pathways that lead to TGA.

## Conclusions

TGA is a rare, benign condition that causes anterograde and retrograde memory loss. Given that there are a lot of theories behind the etiology and pathophysiology, clinicians should be open to a presentation that shows a normal physical examination and no medical history. Efforts should be made to rule out other morbid conditions. In addition, where imaging is concerned, including these distinct features as part of the diagnostic criteria can help to rule out other diagnoses.
